# A case report of breast Rosai-Dorfman disease and a literature review

**DOI:** 10.3389/fonc.2025.1474931

**Published:** 2025-02-13

**Authors:** Yuan Lin, Ya-Wen Wang, Li-Xin Li, Zhong-Qi Qiao, Xu Chen, Yan-Duo Chen, Can Liu, Kai Zhang

**Affiliations:** ^1^ Department of Breast Surgery, General Surgery, Qilu Hospital of Shandong University, Jinan, Shandong, China; ^2^ Department of General Surgery, Binzhou Second People’s Hospital, Binzhou, Shandong, China; ^3^ Department of Pathology, Qilu Hospital of Shandong University, Jinan, Shandong, China; ^4^ Department of Pediatric Surgery, Central Hospital Affiliated to Shandong First Medical University, Jinan, Shandong, China

**Keywords:** breast, Rosai-Dorfman disease, extranodal RDD, pathological diagnosis, surgical treatment

## Abstract

Rosai-Dorfman disease (RDD) is a rare idiopathic histiocytoproliferative disease that usually affects the lymph nodes of the head and neck, but can also involve extranodal sites such as the skin, sinuses, and soft tissues. Breast RDD is exceedingly rare. It may be clinically and radiographically similar to neoplastic and non-neoplastic diseases. We report a case of breast RDD in a 68-year-old female patient and describe the clinical imaging and pathological features of the patient. The management of extranodal RDD is individualized, and there are no standardized guidelines for treatment. We highlight the importance of considering the diagnosis of extranodal breast RDD, and suggest that surgical resection is an effective way to treat this disease, particularly for single-focal breast lesions with RDD.

## Introduction

Rosai-Dorfman disease (also known as sinus histiocytosis with giant lymphadenopathy, RDD) is a rare non-Langerhans cell histiocytic benign disease of unknown cause. RDD was first reported in 1965 by French pathologist Pierre Destombes, who thought it was a lipid storage disease caused by inflammation (1). Subsequently, in 1966, Azoury and Reed described an unusual case of histiocytic hyperplasia with microscopic features that differed from known histiocytic hyperplasia (2). In 1969, American pathologists Rosai and Dorfman correctly identified the key role of tissue cells in the pathogenesis of the disease, with the most common manifestations being painless cervical lymph node enlargement, and low fever, and further summarized 34 similar cases, officially naming the disease Rosai-Dorfman disease (3–5). Early understanding of the disease focused on its impact on lymph nodes, with patients typically presenting with painless, unilateral or bilateral lymphadenopathy, often accompanied by low-grade fever, weight loss, and night sweats. With further research, it has been found that Rosai-Dorfman disease is not limited to lymph nodes and can also affect multiple organs, including the skin, bones, respiratory tract, and gastrointestinal tract. Breast involvement is a very rare manifestation, with no more than 50 cases of breast RDD reported to date (6). The clinical manifestations and imaging findings of breast RDD are often non-specific, resembling either tumorous or non-tumorous conditions, which reflects the importance of pathological analysis in diagnosis. Biopsy typically reveals characteristic pathological features, including patchy proliferation of foamy histiocytes with emperipolesis. Immunohistochemically, the disease is marked by infiltration of large histiocytes and lymphoplasmacytic cells. The histiocytes show positive staining of S100 and CD68, commonly displaying emperipolesis.

The vast majority of cases of Rosai-Dorfman disease are considered to be benign reactive proliferations, but a small number of cases may present as chronic, persistent diseases, and in rare instances, may even progress to malignancy. The exact pathogenesis remains unclear, but studies have suggested it may be closely related to immune system dysregulation and certain genetic factors. Recent research has revealed that the development of Rosai-Dorfman disease (RDD) may be closely linked to specific genetic mutations, such as BRAF, KRAS, and NRAS involving in the abnormal activation of the MAPK/ERK signaling pathway (7). Studies have shown that the BRAF V600E mutation is clinically relevant in some RDD patients, especially in relation to chronicity, recurrence, and resistance to conventional treatments (8). Additionally, the abnormal expression of certain immune regulatory factors, such as IL-10 and TNF-α, are also believed to play a key role in the immune pathological mechanisms of RDD (9). RDD is, therefore, associated with multiple gene mutations that exhibit different distributions across different subtypes of the disease. In addition, these genetic mutations are also associated with prognosis, as specific mutations (such as BRAF or KRAS) often suggest a more aggressive disease course and poorer treatment outcomes (10).

Although there are currently no treatment guidelines for RDD, several options ranging from close observation to surgical resection have been proposed (11). In recent years, molecular studies targeting specific mutations have laid the foundation for personalized treatment and precision medicine in RDD, offering important insights for the development of novel targeted therapies and immunotherapies aimed at improving patient prognosis. Here we describe a patient with the typical clinical characteristics of breast RDD, and discuss the clinical imaging findings, auxiliary findings, and course of treatment and follow-up.

## Clinical data

A 68-year-old female with “a lump in the left breast for six months” was admitted to the hospital. The patient had an incidentally discovered lump in the left breast, located in the upper outer quadrant of the left breast, about the size of an “apricot”, without tenderness, red or purple skin on the surface, nipple bleeding, discharge, skin redness or swelling. The patient previously did not receive diagnosis and treatment of the breast lump, but recently noticed that the mass gradually increased to the size of an “egg”. Also the red and purple range of the skin on the surface of the mass increased, so she was admitted to our hospital.

She had a history of hypertension for two years, managed with oral nicardipine (10 mg once daily), and newly diagnosed elevated blood glucose (6.49 mmol/L), controlled by diet. She also had a history of surgical treatment for a “left upper limb fracture”, with good postoperative recovery. Her obstetric history included five pregnancies, with two live births and no history of dysmenorrhea. She had no history of food or drug allergies and denied a family history of breast tumors and other malignancies.

**Figure 1:**
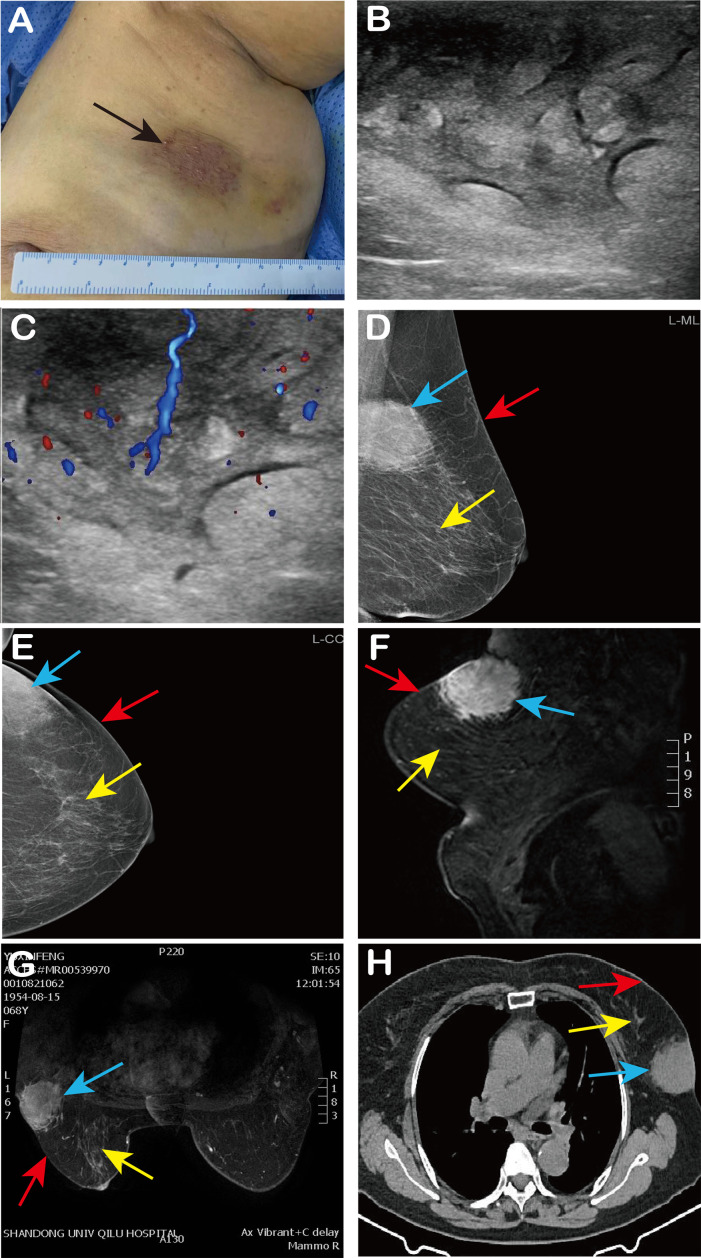
Patient’s clinical findings and imaging findings. **(A)** We observed red and purple skin changes on the surface of the mass (black arrow); **(B, C)** Ultrasound showed an echo-enhanced mass of 8 cm × 5 cm × 3 cm in the left breast, with cobblestone changes and abundant internal blood flow signals; **(D, E)** Left breast mammography (MLO, CC) revealed an asymmetric dense shadow in the upper outer quadrant of the left breast; **(F, G)** MRI of the breast indicated an abnormal signal in the glandular tissue at the posterior edge of the upper outer quadrant involving the skin; **(H)** CT shows a soft tissue density mass in the left breast with spiculated margins and connection to the skin. In Figures **(D–H)**, the blue arrow indicates the lump, the red arrow indicates the breast skin, and the yellow arrow indicates the breast tissue.

Physical examination revealed a hard, irregularly shaped, non-mobile mass of approximately 9 cm × 7 cm in size near the axilla in the upper outer quadrant of the left breast. The mass adherent to the skin had an unclear boundary, an irregular surface, and showed overlying skin discoloration (**Figure 1A**).

Pre-admission ultrasound revealed significant thickening of the skin and subcutaneous soft tissues in the upper outer quadrant of the left breast, with increased echogenicity and a cobblestone appearance. The lesion measured approximately 8 cm × 5 cm × 3 cm and exhibited rich blood flow signals, suggesting localized skin and subcutaneous soft tissue edema (**Figures 1B, C**). Mammography revealed an asymmetric dense shadow in the upper outer quadrant of the left breast, classified as BI-RADS 4B (**Figures 1D, E**). Magnetic Resonance Imaging (MRI) of the breast indicated an abnormal signal in the glandular tissue at the posterior edge of the upper outer quadrant, involving the skin, with a suspicion of malignancy classified as BI-RADS 4C. Differential diagnosis included atypical low-grade angiosarcoma and cutaneous Rosai-Dorfman disease (**Figures 1F, G**). Chest Computed Tomography (CT) revealed a soft tissue density mass in the outer quadrant of the left breast, with spiculated margins and connection to the skin (**Figure 1H**). After admission, the patient underwent fasting peripheral venous blood collection in the morning. Blood routine based on whole blood, blood biochemistry, liver and kidney function and infectious disease analysis on serum, and blood coagulation analysis on plasma, were tested. The results of all these laboratory tests were within the normal range.

**Figure 2:**
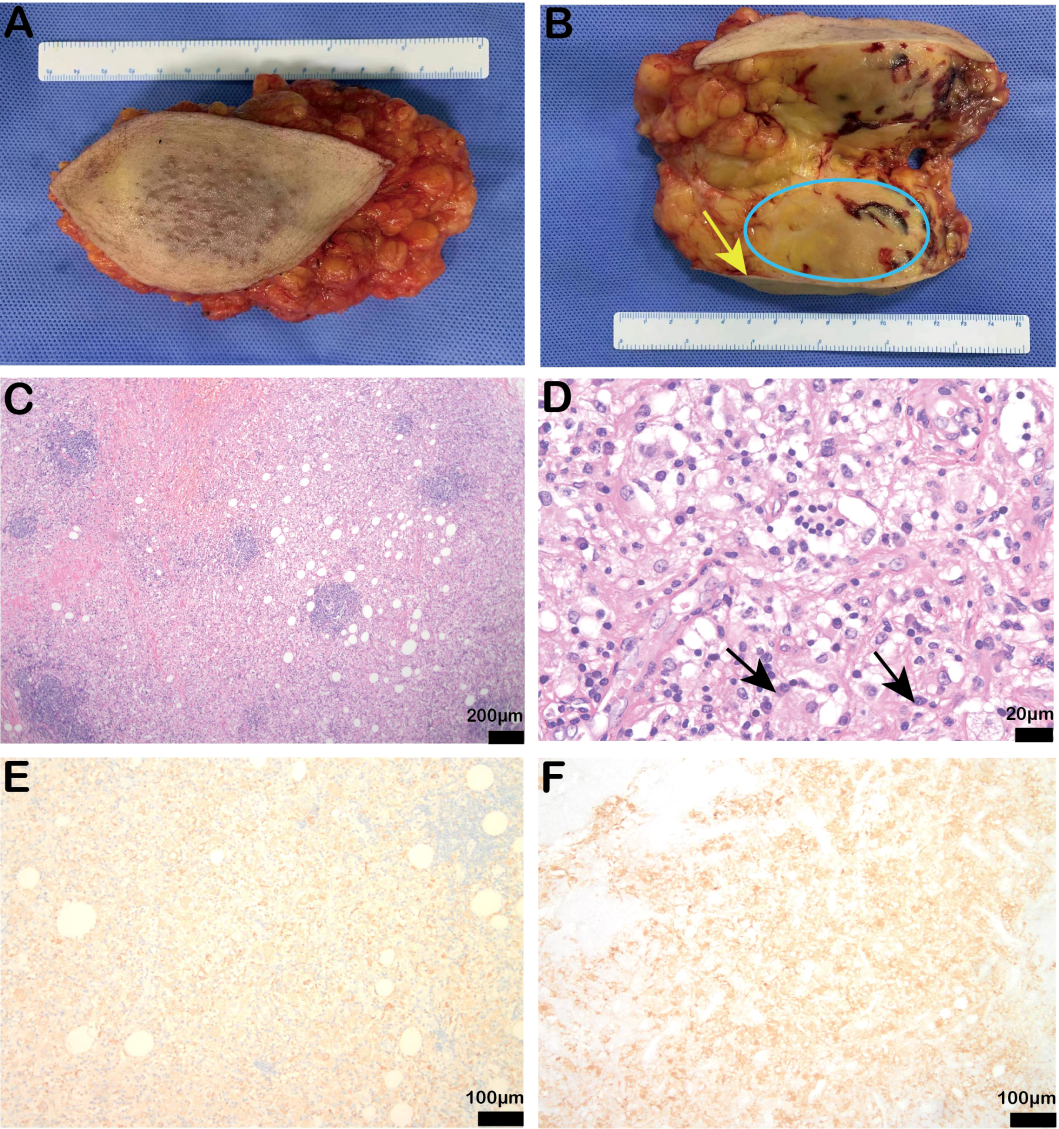
Postoperative specimen findings and pathological results of the patient. **(A, B)** Intraoperative specimen and profile (yellow arrow indicates breast skin, blue coil indicates breast mass); **(C, D)** Stained bands and the hyperstained bands were arranged alternately, and the light-stained areas were spindled to polymorphic histiocytes with large volumes and varying numbers of lymphocytes and plasma cells in the cytoplasm. The arrows showed the protrusion phenomenon [original magnification of H & E: **(C)** × 40, scale bar = 200μm; **(D)** × 400, scale bar = 20μm]; **(E, F)** Immunohistochemistry showed positive CD68 (IHC: × 100, scale bar = 100μm) and partial positive S100 expression (IHC: × 100, scale bar = 200μm).

Following admission, a biopsy of the left breast mass was performed. Pathology results indicated that collagen fiber hyperplasia with a large amount of foam-like tissue and plasma cell infiltration was present. Immunohistochemistry revealed S-100 (+), CK (-), CD31 (-), CD30 (+), LK (-), CD38 (plasma cells +), ERG (-), CD3 (T cells +), CD68 (histiocytic cells +), CD20 (B cells +), and a Ki-67 positivity rate of 5%. Thus Rosai-Dorfman disease was considered. The patient then underwent quadrantectomy of the left breast (**Figures 2A, B**). Postoperative routine pathology confirmed Rosai-Dorfman disease (**Figures 2C-F**), and immunohistochemistry revealed CK (-), CD68 (+), S-100 (+), CD38 plasma cells (+), CD138 plasma cells (+), CD3 lymphocytes (+), CD31 blood vessels (+), CD34 (-), and CD1a scattered weakly (+), and the Ki-67 positivity rate was 5%.

A one-year postoperative follow-up indicated that the patient was well with good wound healing and no recurrence (**Supplementary Figure S1**).

## Discussion

According to the revised classification by the Histiocyte Society, Rosai-Dorfman disease is part of group R of histiocytosis, which includes familial RDD, classic RDD, extranodal RDD, tumor-associated RDD, RDD associated with immune disorders, and various other types of histiocytosis (12). Over 40% of patients have extranodal RDD, commonly affecting sites such as the nasal cavity, skin, orbits, bones, and central nervous system (13). Some studies suggest that RDD may result from immune dysregulation or infection, either through cell-mediated immune disorders or in association with infections by varicella-zoster virus, herpes simplex virus, Epstein-Barr virus, cytomegalovirus or HIV (14). Classic RDD commonly presents with painless cervical lymphadenopathy, with symptoms such as fever, night sweats and weight loss (3, 5). When extranodal RDD occurs in the nasal cavity, the main clinical manifestations are nasal obstruction and nasal mass (15); when it occurs in the skin, it could manifest as papulonodular lesions, sclerotic plaques, tumor-like lesions, acneiform lesions, xanthomatous rash, and etc. (16).

There are few reports on breast RDD in the English literature. We searched and reviewed previously published literature, including case reports and case series. As shown in **Table 1**, we collected 48 cases of RDD involving breast and related information (6, 17–30). Breast RDD occurs mostly in women, but there are reports in men as well (22, 27). Patients with RDD confined to the breast generally present with a local, slow-growing, painless mass, which is hard in texture and poorly demarcated. Radiological examination may not clearly distinguish RDD from breast cancer, with mammograms possibly showing single or multiple poorly-defined masses without calcifications, and ultrasound showing hypoechoic features (30–32). In our case, the patient presented as an echo-enhanced mass in the upper outer quadrant of the left breast, with significant thickening of the outer glandular skin and subcutaneous soft tissue. Here the RDD cases, originated from the sinus histiocyst of the breast, affected the breast skin. Although the disease does not originate from the breast epithelial cell, the lesions do stem from the component the breast. Considering the imaging and pathological evidence of breast invasion, we named this case as breast RDD.

**Table 1:** Information of typical cases of breast RDD reported previously in the literature.

Reference	Case No.	Age	Gender	Clinical presentation	Breast Side	Extramammary involvement	Diagnostic modality	Treatmnet of breast lesion	Follow-up (mouths)	Outcome
Green 1997 (17)	7	median 46 (range 15-84)	F	Breast mass	Right - 3, Left - 1, Bilateral - 2, N/A - 1	yes - 3, no - 4	Core biopsy	Excisoonal biopsy	6-60 2 N/A	No recurrence - 2 Systemic RDD Deceased - 1 N/A - 4
Hummel 1999 (18)	1	52	F	Breast mass	Left	yes	FNA	Excisoonal biopsy	18	No recurrence
Bansal 2010 (19)	1	35	M	Breast mass	Right	no	FNA	Untreated	18	No recurrence
Tenny 2011 (20)	1	64	F	Breast mass	Right	yes	Core biopsy	Excisoonal biopsy	6	Recurrence
Mantilla 2016 (21)	2	59,67	F	Breast mass	Left - 1, N/A - 1	N/A	Core biopsy	Untreated - 1, N/A - 1	36 -1 N/A - 1	No recurrence - 1 N/A - 1
El-Attrache 2018 (22)	1	55	M	Breast mass	Right	no	Core biopsy	Excisoonal biopsy	36	Recurrence
Hoffmann 2019 (23)	22	median 54 (range 37-71)	F- 18,M- 4	Breast mass or detectd on MG	N/A	no	Core biopsies - 4, Excisional biopsies/lumpectomies - 17, Mastectomy - 1	N/A	6-72 24 72 N/A	No recurrence - 4 Recurrence - 1 Recurrence - 1 N/A - 16
Shetty 2020 (24)	4	median 58 (range 43–69)	F	Breast mass	Right	no	Core biopsies - 1, Excisional biopsy - 1, Reduction Mastectomy - 1, N/A - 1	Reduction Mammoplasty - 1, Partial mastectomy - 1, Excisional biopsy - 1, Excisional biopsy - 1	12-168 - 3 N/A - 1	No recurrence - 3 N/A - 1
Battle 2021 (25)	1	49	F	Breast mass	Right	no	Core biopsy	Untreated	12	No recurrence
Iancu 2021 (6)	1	63	F	Breast mass	Left	no	Core biopsy	Untreated	12	No recurrence
Reddy 2021 (26)	2	36,58	F	Breast mass or detectd on MG	Left	yes - 1, no - 1	Excisional biopsy	Excisional biopsy	12-24	No recurrence
Nguyen 2023 (27)	1	58	M	Breast mass	Left	no	Core biopsy	Excisoonal biopsy	24	No recurrence
Prakashchandra Suthar 2024 (28)	1	55	F	Breast mass	Left	yes	Core biopsy	Excisoonal biopsy	N/A	N/A
Zhi Qing Choo 2021 (29)	2	52,37	F	Breast mass or detectd on CT	Left	no	Core biopsy	Excisoonal biopsy - 1, Untreated	48 N/A	No recurrence - 1 N/A
Sumner 2022 (30)	1	59	F	Abnormal detectd on MG	Bilateral	no	Core biopsy	Untreated	N/A	N/A

F, Female; M, Male; MG, Mammography; CT, Computed Tomography; N/A, not available; FNA, fine needle aspiration; RDD, Rosai-Dorfman disease; - “n”, stands for number of cases.

RDD most commonly affects the superficial and dermal layers, consistent with its frequent extranodal presentation in the skin. Cutaneous RDD is a rare manifestation of RDD limited to the skin and subcutis (33, 34). Two dermatologists who collected data from patients diagnosed with cutaneous RDD found that the lesions typically present as non-specific, asymptomatic red-brown to yellow papules, nodules, or plaques, which may be localized or disseminated (35). The lesions may remain confined to the skin and dermis, or appear as protruding nodules or masses on the skin surface or in the subcutaneous tissues. For instance, a 55-year-old woman diagnosed with cutaneous RDD presented with multiple tubercles protruding from the skin of her trunk and extremities (36). Similarly, a 14-year-old girl presented with a tender, active subcutaneous mass in her left arm that lasted three years, which was surgically removed and confirmed as cutaneous RDD (37). In our case, the breast lesions invaded the skin, causing redness, subcutaneous edema, and a palpable lump in the subcutaneous breast tissue. The unusual presentation of a breast lump in an elderly woman may raise concern for breast malignancy, which is atypical for RDD (38).

Invasive breast cancer and lymphoma may also present as masses with increased echogenic borders. The imaging characteristics of RDD can resemble those of breast carcinoma, particularly invasive ductal carcinoma (IDC), which often presents as a poorly defined mass. However, IDC typically arises from the fibroglandular tissue and often shows microcalcifications on mammography, which is less common in RDD. Similarly, fibrocystic changes or benign breast tumors may appear as well-circumscribed masses on imaging, though they are often more mobile and associated with benign clinical signs. Extranodal non-Hodgkin lymphoma (NHL) may also present as a breast mass, and its imaging characteristics may overlap with RDD. However, lymphoma typically presents with more systemic symptoms, such as fever, weight loss, and night sweats, and biopsy would reveal atypical lymphoid cells rather than the “emperipolesis” characteristic of RDD. Additionally, skin abscesses or infected skin appendages may show similar radiological features, but these are usually associated with acute inflammatory signs such as warmth, redness, and tenderness, which distinguishes them from the more indolent nature of RDD. Diagnosis of RDD is typically confirmed by core needle biopsy, which demonstrates emperipolesis (the engulfment of intact lymphocytes by histiocytes) and is immunohistochemically positive for S-100 protein (31).

RDD is a self-limiting benign disease with a good prognosis, and some cases have been reported to resolve spontaneously (39). There are no definitive treatment guidelines for RDD worldwide, and there is still controversy over the treatment. The treatment of RDD should be individualized. For patients with simple lymph node type or skin-only asymptomatic RDD, observation and close follow-up are sufficient; for patients with solitary extranodal lesions or RDD causing local organ compression, surgical excision of the lesion is recommended; for patients with symptomatic lymph node or cutaneous RDD, as well as those with multifocal, inoperable extranodal lesions or systemic, intractable disease, systemic pharmacotherapy-primarily corticosteroids-should be considered (11). Some studies have shown that radiotherapy, chemotherapy, and immunomodulatory therapy are also effective methods for controlling and treating RDD (31). Although surgery is typically limited to biopsy, surgical resection can be curative for localized disease (**Table 1**). Most patients undergo surgical resection at the time of diagnosis and do not experience recurrence during follow-up (40). In this case, the patient also underwent surgical resection, and there was no recurrence after one year, indicating that surgical resection is an effective treatment for breast RDD.

We report a rare case of a 68-year-old female patient with breast RDD. Based on the clinical and radiological finding, this tumor is easy to misdiagnose as a breast tumor. However, tissue pathology, as the gold standard for RDD diagnosis confirmed the disease through core needle biopsy and postoperative routine pathology. This case highlights the variability in the presentation of the disease and the need to recognize the clinical and radiological variability of breast RDD patients, the importance of diagnosis, and the options for treatment methods. Due to the rarity of breast RDD, further research is required to better understand its pathogenesis and develop standardized management guidelines.

## Data Availability

The original contributions presented in the study are included in the article/**Supplementary Material**. Further inquiries can be directed to the corresponding author.

## References

[1] Destombes P . Adenitis with lipid excess, in children or young adults, seen in the Antilles and in Mali. (4 cases). Bull La Societe Pathol Exotique Ses Filiales. (1965) 58:1169–75. 5899730

[2] Azoury FJ Reed RJ . Histiocytosis. Report of an unusual case. New Engl J Med. (1966) 274:928–30. doi: 10.1056/NEJM196604282741702 5908885

[3] Rosai J Dorfman RF . Sinus histiocytosis with massive lymphadenopathy. A newly recognized benign clinicopathological entity. Arch Pathol. (1969) 87:63–70. 5782438

[4] Symss NP Cugati G Vasudevan MC Ramamurthi R Pande A . Intracranial Rosai Dorfman Disease: report of three cases and literature review. Asian J Neurosurg. (2010) 5:19–30. 22028755 PMC3201083

[5] Rosai J Dorfman RF . Sinus histiocytosis with massive lymphadenopathy: a pseudolymphomatous benign disorder. Analysis of 34 cases. Cancer. (1972) 30:1174–88. doi: 10.1002/1097-0142(197211)30:5<1174::AID-CNCR2820300507>3.0.CO;2-S 5083057

[6] Iancu G Gica N Mustata LM Panaitescu AM Vasile D Peltecu G . Rosai-dorfman disease: breast involvement-case report and literature review. Med (Kaunas Lithuania). (2021) 57:1167. doi: 10.3390/medicina57111167 PMC8624438 34833385

[7] Garces S Medeiros LJ Marques-Piubelli ML Coelho Siqueira SA Miranda RN Cuglievan B . Cyclin D1 expression in Rosai-Dorfman disease: a near-constant finding that is not invariably associated with mitogen-activated protein kinase/extracellular signal-regulated kinase pathway activation. Hum Pathol. (2022) 121:36–45. doi: 10.1016/j.humpath.2021.12.013 34995673

[8] Moen FM Youssef MM Shukla M Nierodzik ML Mayerhoefer ME Park C . BRAF V600E mutation and high expression of PD-L1 in Rosai-Dorfman disease: case report and review of the literature. J Hematopathol. (2024) 17:183–9. doi: 10.1007/s12308-024-00611-9 PMC11635026 39592527

[9] Cai Y Shi Z Bai Y . Review of rosai-dorfman disease: new insights into the pathogenesis of this rare disorder. Acta Haematol. (2017) 138:14–23. doi: 10.1159/000475588 28614806

[10] Pai P Nirmal A Mathias L Jain S Shetty MG Sundara BK . Molecular mutations in histiocytosis: A comprehensive survey of genetic alterations. Mol Biotechnol. (2025) 67:438–55. doi: 10.1007/s12033-024-01072-2 PMC11711569 38376733

[11] Abla O Jacobsen E Picarsic J Krenova Z Jaffe R Emile JF . Consensus recommendations for the diagnosis and clinical management of Rosai-Dorfman-Destombes disease. Blood. (2018) 131:2877–90. doi: 10.1182/blood-2018-03-839753 PMC6024636 29720485

[12] Emile JF Abla O Fraitag S Horne A Haroche J Donadieu J . Revised classification of histiocytoses and neoplasms of the macrophage-dendritic cell lineages. Blood. (2016) 127:2672–81. doi: 10.1182/blood-2016-01-690636 PMC5161007 26966089

[13] Bruce-Brand C Schneider JW Schubert P . Rosai-Dorfman disease: an overview. J Clin Pathol. (2020) 73:697–705. doi: 10.1136/jclinpath-2020-206733 32591351

[14] Paulli M Bergamaschi G Tonon L Viglio A Rosso R Facchetti F . Evidence for a polyclonal nature of the cell infiltrate in sinus histiocytosis with massive lymphadenopathy (Rosai-Dorfman disease). Br J Haematol. (1995) 91:415–8. doi: 10.1111/j.1365-2141.1995.tb05313.x 8547085

[15] Ribeiro BN Marchiori E . Rosai-Dorfman disease affecting the nasal cavities and paranasal sinuses. Radiol Brasileira. (2016) 49:275–6. doi: 10.1590/0100-3984.2015.0167 PMC5073402 27777489

[16] St Claire K Edriss M Potts GA . Cutaneous rosai-dorfman disease: A case report. Cureus. (2023) 15:e39617. doi: 10.7759/cureus.39617 37388601 PMC10300236

[17] Green I Dorfman RF Rosai J . Breast involvement by extranodal Rosai-Dorfman disease: report of seven cases. Am J Surg Pathol. (1997) 21:664–8. doi: 10.1097/00000478-199706000-00006 9199644

[18] Hummel P Waisman J Chhieng D Yan Z Cohen JM Cangiarella J . Fine-needle aspiration cytology of Rosai-Dorfman disease of the breast: A case report. Diagn Cytopathol. (1999) 21:287–91. doi: 10.1002/(SICI)1097-0339(199910)21:4<287::AID-DC12>3.0.CO;2-C 10495325

[19] Bansal P Chakraborti S Krishnanand G Bansal R . Rosai-Dorfman disease of the breast in a male: a case report. Acta Cytol. (2010) 54:349–52. doi: 10.1159/000325050 20518426

[20] Tenny SO McGinness M Zhang D Damjanov I Fan F . Rosai-Dorfman disease presenting as a breast mass and enlarged axillary lymph node mimicking Malignancy: a case report and review of the literature. Breast J. (2011) 17:516–20. doi: 10.1111/j.1524-4741.2011.01131.x 21762247

[21] Mantilla JG Goldberg-Stein S Wang Y . Extranodal rosai-dorfman disease: clinicopathologic series of 10 patients with radiologic correlation and review of the literature. Am J Clin Pathol. (2016) 145:211–21. doi: 10.1093/ajcp/aqv029 26803323

[22] El-Attrache B Gluck B Heimann A Kapenhas E . A rarity in breast pathology: First recurrent male case of Rosai-Dorfman disease. Int J Surg Case Rep. (2018) 52:137–9. doi: 10.1016/j.ijscr.2018.10.003 PMC6199774 30359898

[23] Hoffmann JC Lin CY Bhattacharyya S Weinberg OK Chisholm KM Bayerl M . Rosai-dorfman disease of the breast with variable igG4+ Plasma cells: A diagnostic mimicker of other Malignant and reactive entities. Am J Surg Pathol. (2019) 43:1653–60. doi: 10.1097/PAS.0000000000001347 31436555

[24] Shetty S Sharma N Booth CN Oshilaja O Downs-Kelly EP McKenney JK . Mammary extranodal rosai-dorfman disease with and without associated axillary lymphadenopathy: insights for practitioners of breast pathology. Int J Surg Pathol. (2020) 28:541–8. doi: 10.1177/1066896920901770 31992097

[25] Battle B McIntire P Babagbemi K Mema E . Extranodal multifocal Rosai-Dorfman disease of the breast: A case report. Clin Imaging. (2021) 71:49–51. doi: 10.1016/j.clinimag.2020.07.012 33171367

[26] Reddy AS Joshi S Popat P Shet TJHPCR . Rare presentations and literature review of Rosai Dorfman disease of the breast. Human Pathol. (2021) 24:200503. doi: 10.1016/j.ehpc.2021.200503

[27] Nguyen T Gutema M Ye J Backenstoss MS . Mammary Rosai-Dorfman disease: Rare benign mimic of breast Malignant neoplasm. J Clin Imaging Sci. (2023) 13:24. doi: 10.25259/JCIS_40_2023 37680249 PMC10481821

[28] Suthar PP Sivakumar A Scaria G Singh JS . Extranodal rosai-dorfman disease of breast mimicker of breast Malignancy. World J Nucl Med. (2024) 23:119–22. doi: 10.1055/s-0043-1760763 PMC11199029 38933064

[29] Choo PZQ Loh AHL Selvarajan S Tan PH Tan VKM Yong WS . Breast-related extranodal Rosai-Dorfman disease presenting as subcutaneous masses with thick hyperechoic rim, with review of the literature. Breast J. (2021) 27:883–6. doi: 10.1111/tbj.v27.12 34467595

[30] Sumner C Salem K Abunimer L Ewaz A Zhang L Monsrud A . Bilateral breast Rosai-Dorfman disease screen detected by mammography. Clin Case Rep. (2023) 11:e6983. doi: 10.1002/ccr3.v11.3 36950663 PMC10025253

[31] Delaney EE Larkin A MacMaster S Sakhdari A DeBenedectis CM . Rosai-dorfman disease of the breast. Cureus. (2017) 9:e1153. doi: 10.7759/cureus.1153 28503389 PMC5426819

[32] Yu KK Briones NF Chan M Ahmed A Stevens E . Rosai-Dorfman disease simulating metastatic breast carcinoma. JAAD Case Rep. (2019) 5:372–4. doi: 10.1016/j.jdcr.2019.02.021 PMC6454097 31008172

[33] Brenn T Calonje E Granter SR Leonard N Grayson W Fletcher CD . Cutaneous rosai-dorfman disease is a distinct clinical entity. Am J Dermatopathol. (2002) 24:385–91. doi: 10.1097/00000372-200210000-00001 12357197

[34] Lu CI Kuo TT Wong WR Hong HS . Clinical and histopathologic spectrum of cutaneous Rosai-Dorfman disease in Taiwan. J Am Acad Dermatol. (2004) 51:931–9. doi: 10.1016/j.jaad.2004.04.030 15583585

[35] Litaiem N Trimech R Daoud Y Gara S Slouma M Jones M . Cutaneous involvement in Rosai-Dorfman disease: clinical and dermoscopic features. Int J Dermatol. (2024). doi: 10.1111/ijd.17570 39526545

[36] Gillam J Desai R Louie RJ Turner SA Wang GY Diaz-Perez JA . Cutaneous Rosai-Dorfman disease with MAP2K1 mutation, initially mimicking an infection with parasitized histiocytes. J Cutaneous Pathol. (2024) 51:942–7. doi: 10.1111/cup.v51.12 39122669

[37] Al-Hashemi RW Aldarraji SS Abdalla T Hasnah S Abu-Dayeh A Telfah HK . The lone lump: cutaneous rosai-dorfman disease as an isolated upper arm lesion. Cureus. (2024) 16:e63542. doi: 10.7759/cureus.63542 39086775 PMC11289357

[38] Parkash O Yousaf MS Fareed G . Rosai-Dorfman’s disease, an uncommon cause of common clinical presentation. JPMA J Pakistan Med Assoc. (2019) 69:1213–5. 31431783

[39] Pulsoni A Anghel G Falcucci P Matera R Pescarmona E Ribersani M . Treatment of sinus histiocytosis with massive lymphadenopathy (Rosai-Dorfman disease): report of a case and literature review. Am J Hematol. (2002) 69:67–71. doi: 10.1002/ajh.10008 11835335

[40] Wang Q Bradley K Zhang M Li S Li X . Rosai-Dorfman disease of the breast: a clinicoradiologic and pathologic study. Hum Pathol. (2023) 141:30–42. doi: 10.1016/j.humpath.2023.08.009 37673345

