# Supplementary figures and images for: A case report of breast Rosai-Dorfman disease and a literature review
Source: Front Oncol. 2025 Feb 13;15:1474931. doi: 10.3389/fonc.2025.1474931 (PMC11865211; doi:10.3389/fonc.2025.1474931)

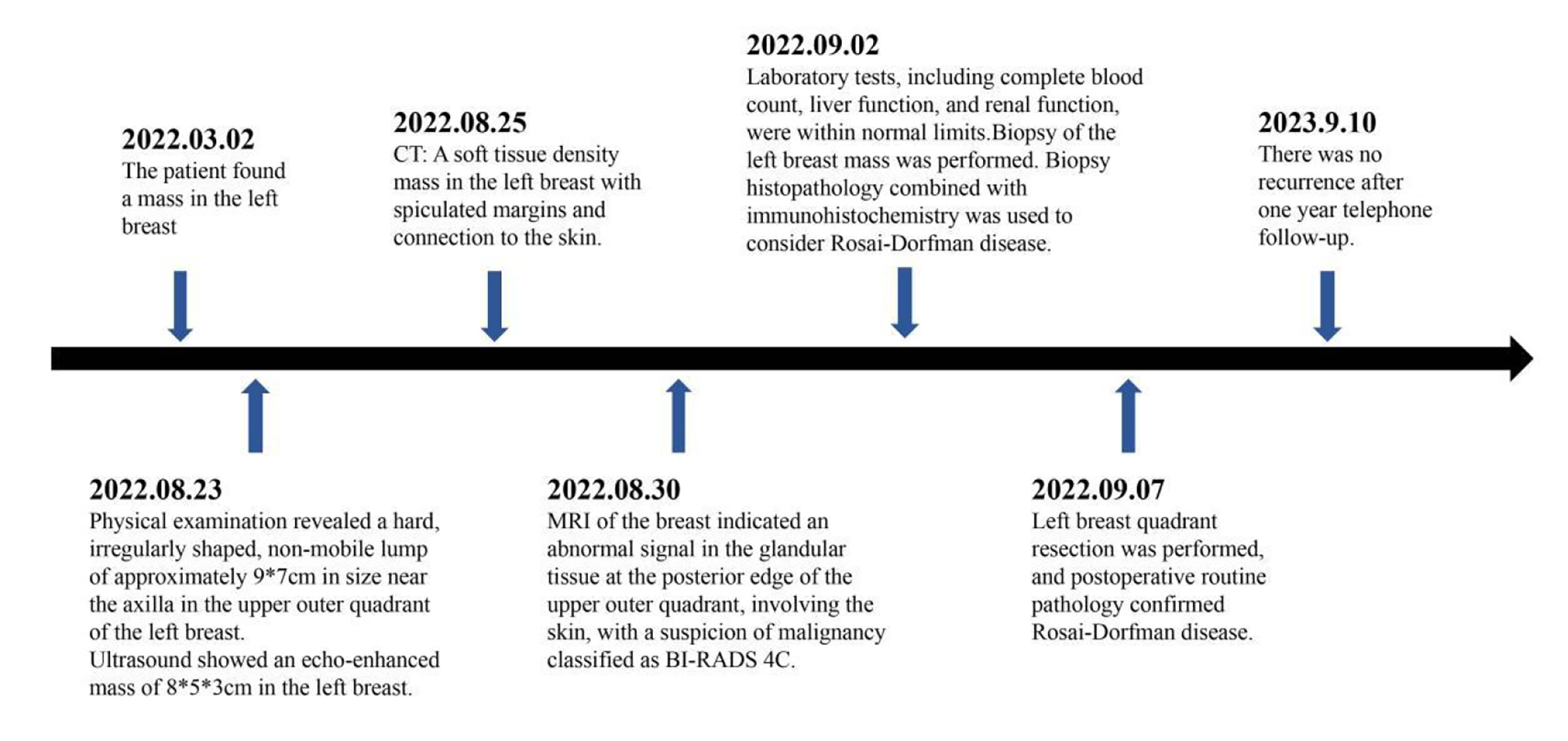

Supplement: Supplementary file 1 [file Image1.tif]
